# Peer support meeting of people with dementia: a qualitative descriptive analysis of the discussions

**DOI:** 10.1186/s12877-023-04329-8

**Published:** 2023-10-09

**Authors:** Fumiko Miyamae, Mika Sugiyama, Tsutomu Taga, Tsuyoshi Okamura

**Affiliations:** Research Team for Promoting Independence and Mental Health, Tokyo Metropolitan Institute for Geriatrics and Gerontology, 35-2 Sakae-Cho, Itabashi-ku, Tokyo, 173-0015 Japan

**Keywords:** Compassion, People with dementia, Peer support meeting, Narrative, Qualitative descriptive analysis.

## Abstract

**Background:**

Dementia cafés for people with dementia and their caregivers are promoted in national dementia policies. The effect of dementia cafés on people with dementia has been reported through narratives of caregivers who participated the dementia cafés. However, evidence derived from the data, which included only people with dementia, is sparse. The aim of this study is to analyze the narratives of people with dementia in peer support meetings in Tokyo where only people with dementia participate, i.e., caregivers were not present.

**Methods:**

People with dementia and older people with subjective cognitive impairment were recruited in our community-based participatory research centre. Based on the qualitative descriptive approach, we conducted a thematic analysis of the field notes, which was made through ethnographical observation of the meetings.

**Results:**

Twenty-five meetings were held from November 2018 to March 2020. The cumulative total number of participants was 196. First, the symptomatic problems related to living with dementia were mentioned, which were collectively named under the overarching category of ‘Experience of living with dementia.’ Second, questions and solutions to the various symptoms were discussed, which were named the ‘Quest of Symptoms.’ Third, we noted the narrative that reflected on daily life, feelings, and the life that one has led, which were named ‘Life story.’ Fourth, we noted narratives of how symptoms have improved and their world has expanded, which were named ‘Hope.’ Fifth and most importantly, narratives about compassion for people with dementia in the past and future, as well as for people of the same generation, were discussed, which were named ‘Compassion.’

**Conclusions:**

The lived experiences of people with dementia were revealed. Participants noted they were not just being cared for but exchanging information and exploring the symptoms; in other words, they were resilient. Furthermore, more positive aspects concerning living with dementia were discussed, such as ‘Hope’ and ‘Compassion.’ Further research concerning the discourse of people around the participants is necessary to evaluate the situation from multiple perspectives.

## Introduction

For people experiencing difficulties due to illness or disabilities, meeting people with similar difficulties can encourage and motivate them to continue living. Encountering others who share similar experiences living with dementia can be empowering. Promoting such activities is expressed clearly in national dementia policies. In the UK national dementia strategy in 2009 [1], which pioneered national dementia strategies, objective five was ‘Develop structured peer support and learning networks’. In Japan’s dementia strategy, which was established in 2015 [2], the word peer support was not used, but a similar idea was expressed as a ‘dementia café.’ However, caregivers often receive more emphasis than individuals with dementia in the Japanese national dementia strategy. In the fifth pillar, it states ‘…to promote dementia cafés where people with dementia and their families can mutually share information and understand each other with local people and specialists to reduce the burden on caregivers of people with dementia’. This trend has continued, with more attention paid to family caregivers. In 2019, the government’s Ministerial Council on the Promotion of Policies for Dementia Care unveiled the Framework for Promoting Dementia Care (3), which stated ‘the burden on family caregivers will be reduced through dementia cafés, family classes, and peer activities among family care givers’.

Several European countries have Meeting Centre Support Programmes (MCSPs), which have been successful. The program originated in the Netherlands and provided locally tailored post-diagnosis support for people with dementia and their family carers. An international multi-center effect study reported that the program showed well-being and health benefits for participants [4].

The effect of dementia cafés on caregivers has been reported. According to Merlo et al. [5], dementia cafés provide significant benefits to caregivers in the management of social and economic problems and lead to better emotional support. However, they did not express benefits for people with dementia. Similarly, Jones et al. [6] reported that caregivers who attended a dementia café reported higher resilience and subjective well-being.

The effect of dementia cafés on people with dementia has been reported to some extent through interviewing caregivers. Greenwood et al. [7] interviewed 11 caregivers of various dementia cafés and reported that dementia cafés are places where people with dementia can feel supported and be themselves. Dow et al. [8] conducted focus group interviews with people with dementia and their caregivers in a dementia café and reported that dementia cafés promote social inclusion, prevent isolation, and improve the social and emotional well-being of attendees. However, this might be due to methodological difficulties, evidence derived from the peer support meeting, which included only the people with dementia is sparse.

Since 2016, a community-based participatory research (CBPR) has been conducted in metropolitan Tokyo (9–10). Based on CBPR, i.e., the most important driving force is building trust with residents and stakeholders, our CBPR includes various community activities. One activity is a peer support group meeting for people with dementia and older people with subjective cognitive impairment. After preparing for the meeting, researchers stay in the background and let older people do what they want during the meeting. We do not strictly distinguish people with dementia from people with subjective cognitive impairment because this process requires a cognitive battery, which might be a barrier to our activity. That is, we did not conduct a mandatory cognitive test before attending the meeting. Because this was included in the large CBPR project, we could record and analyse what was happening. The words in the real world, dementia café, Alzheimer café, memory café, and peer support groups are used without a precise definition. In this manuscript, we use a dementia café to mean a gathering place where people with dementia and their caregivers gather, and we use a peer support meeting as a gathering place without caregivers.

This study explores the discussion at a peer support meeting in our community-based participatory research centre in metropolitan Tokyo. The most important characteristic of the tour study is that caregivers were not at the meeting and that we are analysing the discourse of older people, not caregivers.

## Methods

We adopted a qualitative descriptive approach in this study. This approach is generally used to gain new insights from participants’ reports regarding phenomena about which we know little [11]. A thematic analysis [12] of the interview was conducted. Details are described below.

### Participants

People with dementia and older people with subjective cognitive impairment were recruited from our CBPR field by (1) distributing flyers to nearby hospitals, nursing care facilities, and all community comprehensive support centres, which announced the meeting, and (2) placing notification posters on the bulletin board of the common areas, which are found the ground floor in every building in the housing complex zone.

### Setting

Our CBPR field was Takashimadaira, which is located in the northwest area of Metropolitan Tokyo. It is the largest housing complex district in Japan and was built during the 70s, which was a high-growth period. An administrative corporation currently manages the housing complex.

### Procedure

The peer support meeting was held once a month on the third Saturday of the month at our community-based participatory research centre. The meetings lasted about one hour. Reservations were not required, and there was no charge. Participants could even attend the meeting without disclosing their names or address. Because some people with dementia come with their caregivers and the existence of the caregivers may place pressure on the people with dementia, we also prepared a meeting for caregivers in a different room.

**Fig. 1:**
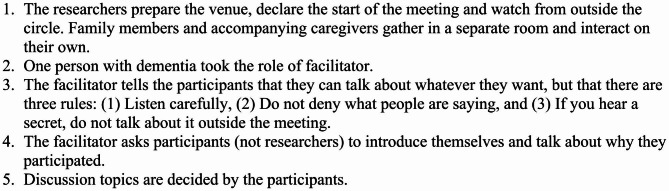
The method for holding the meeting

Before the project’s launch, psychologists, case workers, public health nurses, and leaders of the existing circle for people with early onset dementia gathered and discussed the research aims, participants, recruiting method, and management. Modifications were made as the sessions progressed, and the final structure of the meeting was as shown in Fig. 1.

### Ethics approval and consent to participate

For consent, the recommendations of the Global Alliance for Genomics and Health’s Ageing and Dementia Task Team [13] were followed. This recommendation states that researchers should presume that people with dementia have capacity until they demonstrate otherwise. Participants in this study have the capacity to participate in meetings independently and to communicate with others. All health professionals involved in the project, including the geriatric psychiatrist, were judged to have a consistent willingness and sufficient capacity to participate in the study on an understanding basis. Therefore, consent was not obtained from legally authorized proxies. In addition, participants in this study were not eligible for guardianship, as in Japan, legally authorized representatives cannot be carried out unless the person has severe dementia and is unable to protect their property (known as guardianship).

In this study, when participants attended the meetings, staff told them that they did not have to reveal their names. Therefore, signed consent forms could not be obtained. At the beginning of each meeting, staff explained verbally to participants that they would not be recorded or videotaped, but that they would record what was discussed at the meeting in handwritten notes and that the data would be presented at scientific meetings and published in scientific journals, and cautioned them not to identify themselves personally. Furthermore, the entrance to this CBPR center clearly states that the activities conducted here will be presented at scientific meetings and published in scientific journals and that those who do not wish to will not be part of the presentation if they wish.

The above method of obtaining consent was approved by the Ethics Committee of the Tokyo Metropolitan Institute of Gerontology and Geriatric Medicine (No. 元健イ事3146).

In addition, in order to make the meeting accessible to people with dementia, the guides published by DEEP (14–15) were referred to when preparing the objectives, participants, recruitment methods, and operational considerations. In addition, the leader of an existing early-onset dementia circle, who is also a person with early-onset dementia, participated and supervised all aspects of the meeting.

### Data

#### Quantitative data

**Fig. 2:**
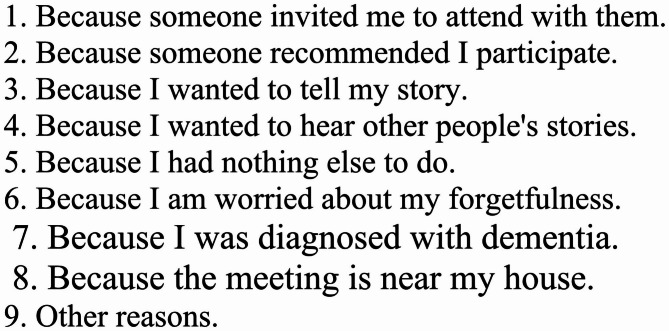
The reason individuals participated in the meeting

For each event, the number of participants was recorded. It is also clear who is participating, so we can see how many times a particular individual attended. Sex and age groups were asked. At each meeting, participants were asked their sex, age, and reason for participating in the meeting via an unmarked questionnaire. Reasons for participation were selected from the options shown in Fig. 2 and multiple responses were allowed.

#### Qualitative data

***1) Ethnographical observations***.

There were always two or three researchers who attended the meeting on-site. Once the meeting started, researchers just observed the meeting. They made field notes about the discourse and behaviour of participants after the meeting.

***2) Staff discussion during the review time***.

Researchers had time to review the meeting afterward, and the discussion in the review was recorded as field notes. The purpose of this review was (1) to continuously refine the actual design of the meeting in the future, and (2) to confirm the participants’ discourses that were missed by the researchers.

***3) Integration of the field notes***.

F.M. always attended the meetings at this site. After the 25th meeting ended, F.M. collected the field notes of the ethnographical observations and staff discussions during the review time from the researchers and re-arranged them in chronological order, which resulted in the dataset for this manuscript. To ensure the reliability of the content of the dataset, researchers who made field notes were asked to review the content. A final agreement was reached on the content.

#### Data analysis

We used a qualitative descriptive approach. This approach is generally used to gain new insights from participants’ discourses regarding phenomena about which we know little [11]. The initial analysis was conducted by F.M., a psychologist with a Ph.D., and T.O., a certified psychiatrist with an MD and Ph.D. They read the dataset several times, became familiar with the content, and generated codes for semantic coherence, keeping in mind the context of the discourse independently. F.M. and T.O. discussed the differences in the code generations until they reached a consensus. We compared the codes based on similarities and differences and grouped them into categories according to their similarities. We further compared the similarities for the categories and grouped them into bridging themes. To increase reliability, psychiatrists, psychologists, and other professionals with expertise in dementia care involved in the project (M.S. and T.T.) met to review the analysis process, discuss it as a team and agreed on coding and interpretation.

## Results

### The meeting description

The meetings were held 25 times between November 2018 and March 2020. The cumulative total number of participants was 196. No adverse events were observed. The number of specific participants was 64; this information was not asked; however, we determined this because we can identify them in person. Figure 3 shows the ratio of the participant’s age group (3a), with a majority of participants aged between 80 and 89 years old, sex ratio (3b) with a majority of women (63.4%), the number of participants in each meeting (3c) with an average of 7.8 participants, and the ratio of each participant according to the number of participations (3d) with 45.3% participation between 2 and 5 times. Figure 4 describes the reasons for each participant’s participation with, 57.1% of the participants coming to the meetings to hear other people’s stories.

**Fig. 3a:**
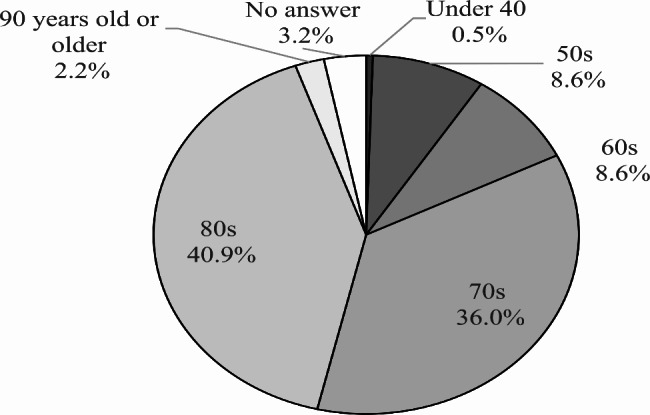
Age Group

**Fig. 3b:**
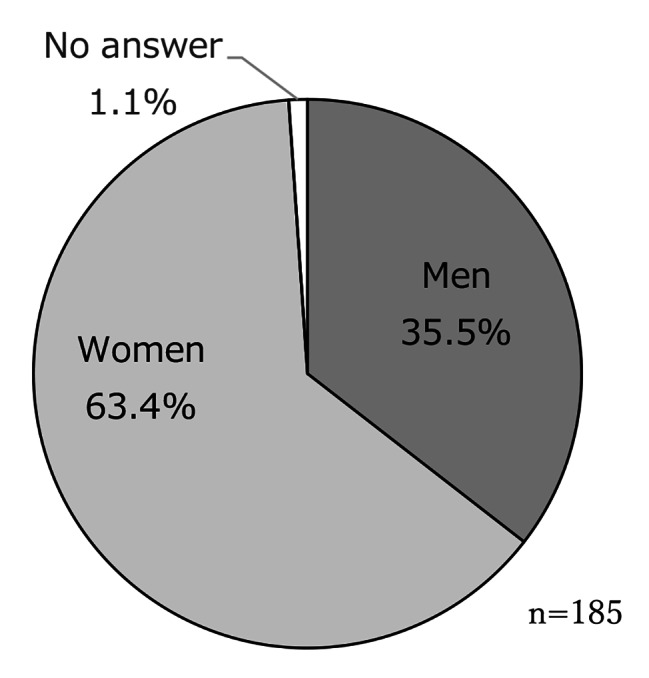
Sex ratio

**Fig. 3c:**
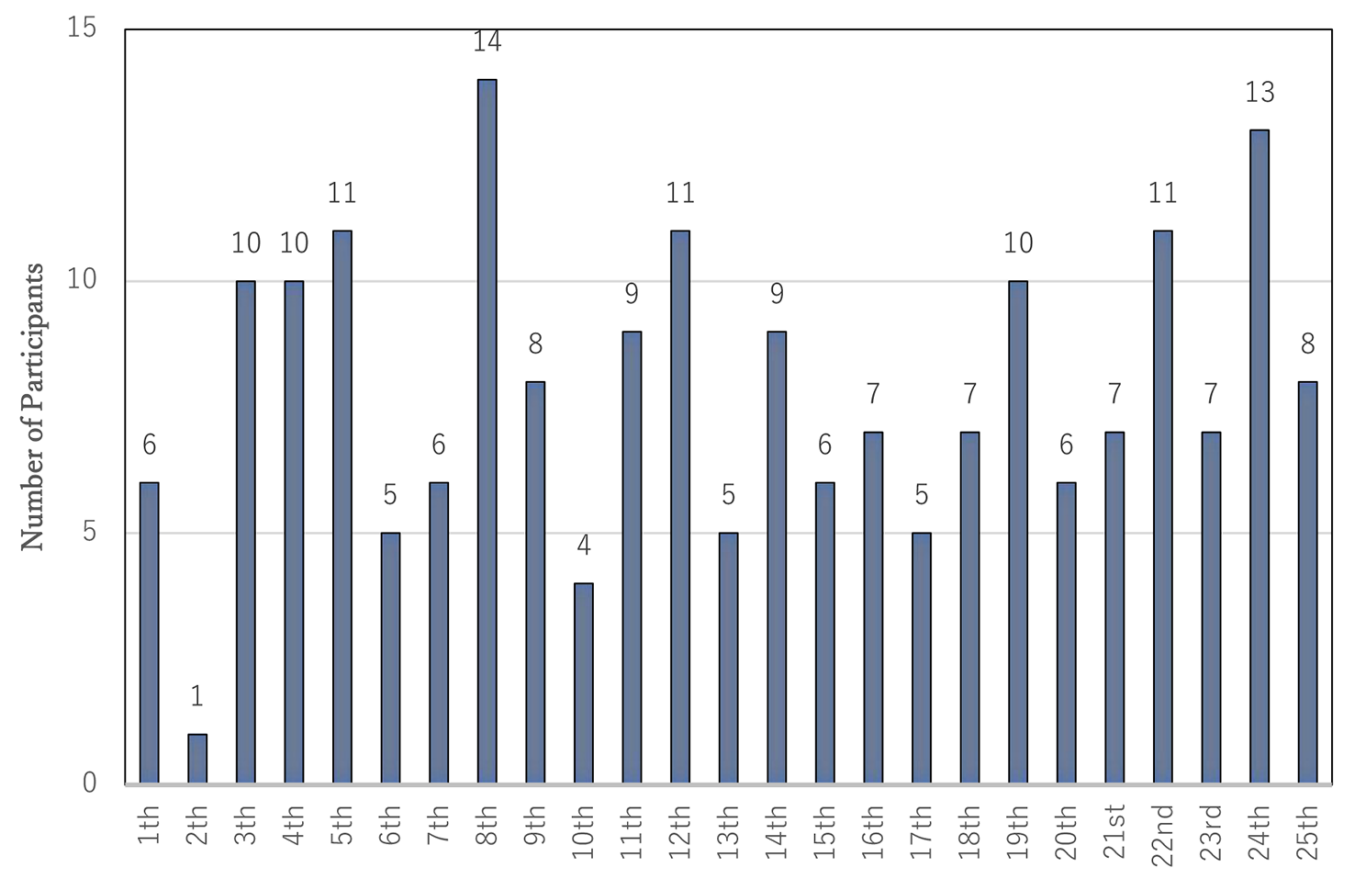
Number people attending meetings during the study period (Nov 2018-Mar 2020)

**Fig. 3d:**
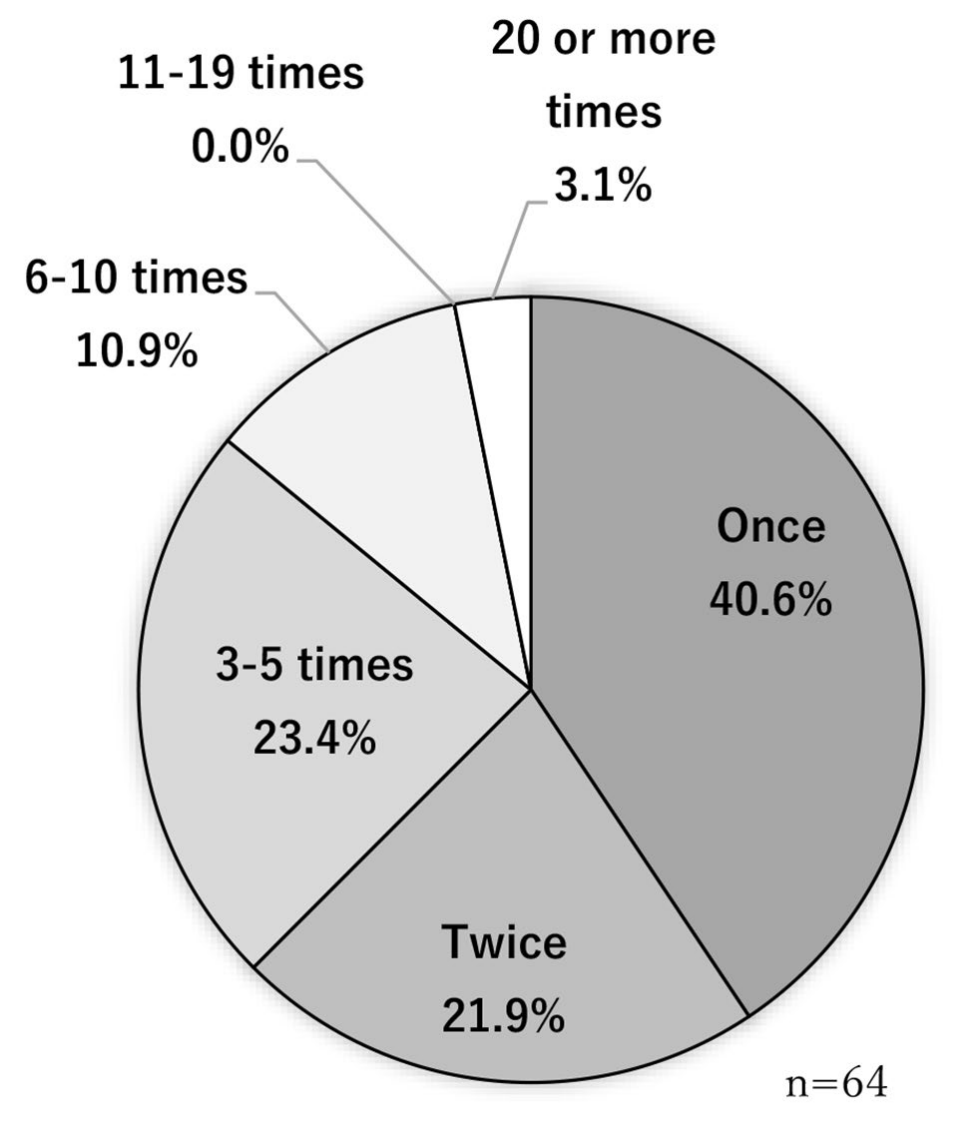
Number of times an individual attended meetings

**Fig. 4:**
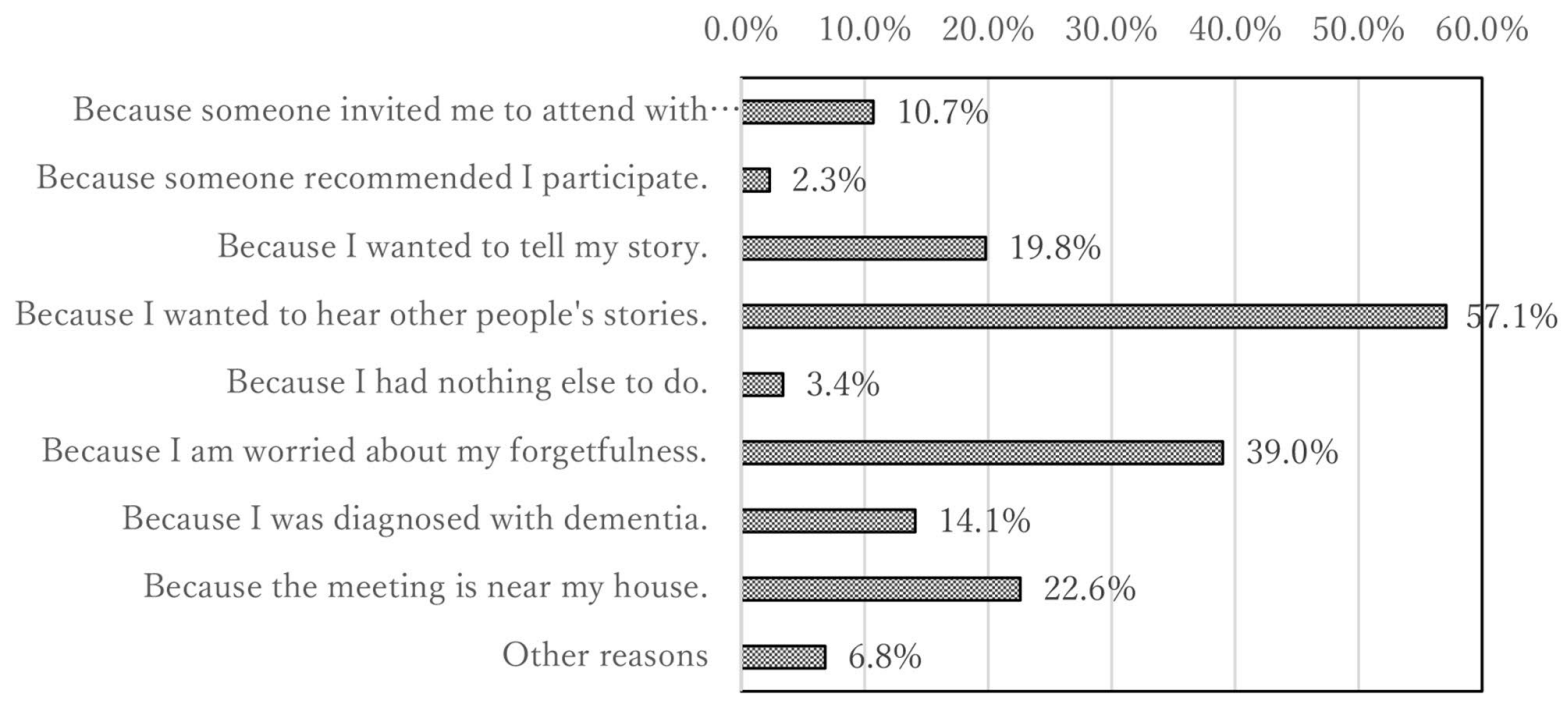
The reasons for meeting participation

### Analysis of what is discussed

The results of the thematic analyses concerning what was discussed are illustrated in Table 1. First, the symptomatic problems related to living with dementia were naturally mentioned, which was expected before the analyses. The contents of symptomatic problems were organized as anxiety, isolation, depression, memory impairment, everyday life problems, lack of self-esteem, hearing impairment, delusion, and cognitive problems. Furthermore, social problems were discussed, which were organized as denial, preparation, conflict with family, mistrust in medicine, and discrimination. These were collectively named under the over-arching category ‘Experience of living with dementia’.

Next, questions about various symptoms were discussed with others, information was exchanged, and solutions were discussed. This was named ‘Quest of Symptoms’. Additionally, narratives reflected on their daily lives and how they felt, and the lives they had led, which were named ‘Life stories’.

Other matters were discussed. They spoke of ‘Hope’ that their symptoms had improved, that they had started volunteer work, and that their world had expanded.

Finally, ‘Compassion’ for people with dementia was discussed. They spoke of compassion for their contemporaries and also of compassion for those who had dementia in the past, i.e., that they did not understand what living with dementia was like at the time, and compassion for those who will have dementia in the future, i.e., that they hope descendants will use their own experiences to live better with dementia in the future.

**Table 1:** What was said in the meeting? thematic analysis of field notes

Overarching category	Category	Subcategory		Significant Narratives Example
Experience of living with dementia	Symptomatic Problems	Anxiety		As I live alone, I am not confident that I would recognize it. So, I have asked a friend to tell my daughter (that I may have dementia). I don’t want to bother people, I don’t want to be like my mother.
		Isolation		Before I knew about this place, I was living like a snail. I don’t have a chance to talk to anyone other than my home care workers, so I tell them the same story over and over again.
		Depression		I was depressed when I was diagnosed as dementia.
		Memory impairment		I can’t remember my cell phone number. I use notes to help my memory, but the moment I try to look at them, if someone says something to me, I forget to look at the notes again.
		Everyday life problems		I forgot to turn off the gas. I once spent half a day in a daze because I couldn’t write Chinese characters.
		Lack of self esteem		The other day I took the wrong bus and got off at a completely different location. Confused by the unfamiliar scenery, it took me 10 min to realize what was going on. I felt sad for myself.
		Healing impairment		My hearing is also weakened, so it is sometimes difficult to understand what people are saying.
		Delusion		I can’t remember where I left the knife. I suspected that the care worker had stolen it…but I tell myself that it cannot be happen and I looked for it, but could not find it. I had a headache because someone irradiated me with radio waves or something.
		Cognitive problems		My brain is always lagging. Sometimes my brain doesn’t work in some climates.
	Denial			I’m ok with the forgetfulness thing, but it’s good to hear everyone’s stories. I do not have (dementia), but I came here for my sister to hear what everyone says.
	Preparation			I try to keep my keys in the same place. I always carry a key card that says I have behavioural variant FTD. I also carry a folded copy of my diagnosis (in case I need to explain it).
	Conflict with family			A was angry that her sister-in-law told her painting teacher that she has dementia without permission. Today, I said I could come alone, but my family insisted that I have to come with them. My family is talking with the doctor right now. They will probably give me some orders later.
	Mistrust in medicine			Even doctors in big hospitals sometimes make mistakes. There is always hit and miss. You have to break your head open to understand. Sometimes doctors don’t know either.
	Discrimination			I could disclose my cancer to my friends, but I could not disclose my dementia. I wanted to hide it, but I can’t hide it anymore. When I told a familiar banker who was visiting my house that I had recently been diagnosed with dementia, he abruptly left without saying hello, and I learned that if you have dementia, you are discriminated against.
Life Stories				I sought out an explanation for why I had such a disease. But there is no use in saying that. My room is uncluttered… Things I don’t want to throw away are piling up. My kids say, “Why don’t you throw it out as soon as possible? I think, “Don’t say it’s easy to throw things away. I think, “Don’t tell me to throw things away so easily.“ They may look like trivial things to others, but they are part of my life.
Quest of symptoms				I don’t know which are symptoms of dementia. Is dementia a disease? As we get older, we lose some of our functions, but when you say it’s a disease… Is there any training to improve dementia? Where should I go first if I have concerns about dementia? I want to know how to interact with a person with dementia.
Hope				Sometimes it’s not okay, but I live with the support of many people. People (with dementia) pay with bills at the cash register. It is preferred by clerks over fumbling around trying to pay with coins. Some aspects are getting better. I am now able to volunteer, work at a tofu shop, and participate in early-onset dementia group. Mr. X disclosed that he had dementia although he is young. This encouraged me. So I disclosed it myself, and it has broadened my world.
Compassion	Past: people with dementia who passed away			My mother had delusions of theft. I now think it was because she wanted to rely on her brother and sister-in-law, who lived with her but were often away from home. Looking back, I said hard things to my mother, but now I realized that there are things you don’t understand until you are that age.
	Present: people with dementia who coexist		Trough gathering	I had never been to these meetings. I was always with my wife. I really wanted someone like a friend. I am very grateful to X for sharing his own experiences with me. I respect X for that.
			Trough helping each other	I joined this group because I wanted to help anyone in need. I noticed that people with dementia were giving advice to others.
			Through making secure place	It saves me to hear everyone talk about it. I can’t do it anymore, but seeing everyone here playing games and playing Go is relaxing.
	Future: people with dementia who will join us in someday			I’m filming myself right now. This is because I want people who will later have dementia to see it.

## Discussion

This study explored what was discussed by older adults, not caregivers, in a meeting held in our community-based participatory research centre in metropolitan Tokyo. As expected, living with dementia was discussed. However, participants were not just being cared for, but they were exchanging information and exploring the symptoms. In other words, they were resilient. Furthermore, more positive aspects concerning living with dementia were discussed such as ‘Hope’ and ‘Compassion’.

In 2015, a research group conducted focus group interviews in a conference room with people with valid dementia diagnoses. The authors of the current study participated in that study, and although there was no direct continuity between the two, they were strongly influenced by it. According to their report, people who had a dementia diagnosis discussed personal support needs, everyday life difficulties, and medical and welfare support [16]. The topics in the current study were very different from those in 2015. This difference may be caused by the setting and participants; the current meeting was held in the CBPR centre in the community where anyone can visit and stay at any time; the current study consisted of residents of the specific community who had dementia or mild cognitive impairment, or even subjective cognitive impairment was not disclosed at all; they talked as the citizens, not patients.

The topic of this study was “Quest of symptoms,“ or trying to understand and manage their own symptoms. According to previous studies on peer support meetings with people with chronic conditions, the topics were reported to be social support, psychological support, practical support, empowerment, condition monitoring and treatment adherence, informational support, behavioural change, encouragement and motivation, and physical training [17]. More specifically, cancer patients tend to talk about encouragement [18].

The topic of “life story” might have a similar effect to ‘Reminiscence’ and ‘Life Review’ which are well-established psychological methods among older people. According to the literature, reminiscence and life review are effective in promoting social interaction, improving mood and self-affirmation, and integrating positive and negative aspects of life (19–20). As far as we searched, compassion was not the major topic in the peer support meetings. One potential reason is the following consideration: Arimitsu et al. [21] compared the characteristics of compassion between two cultures, Japan and the US. They found that in the US, self-compassion had a stronger relationship with positive affect, whereas compassion for others was related to interdependent happiness in Japan. Following their theory, our participants were happy when talking about compassion for people with dementia like themselves, in the past, present, and future.

One possible reason for such a wide variety of topics discussed is that the meeting setting was carefully planned by the experts (Fig. 1). Some participants commented that they felt comfortable speaking in the meetings because the three rules protected them. In this meeting, the person with dementia and their family were separated. This enabled the person with dementia to say what they wanted to say without worrying about their family’s reaction. This method was proposed by a person with dementia [16]. This separation caused no problems because [1] the staff explained to both companies well in advance about the purpose of the meeting, and [2] the staff assured the family that the people with dementia would be well supported by the experts during the period they are separated. Provision of information in order to make the person with dementia and their family members participate with peace of mind is essential to peer support meetings of people with dementia.

### Strengths and limitations

The study’s strength lies in the fact that (1) we analysed what was discussed by older adults, not caregivers; (2) the meeting was held in the CBPR centre in the community where anyone can visit and stay at any time, which resulted in a more natural atmosphere, not a medical research atmosphere.

The limitations of this study were (1) this was the analysis of a single series of peer-support meetings, which was affected by specific characteristics such as location and staff; (2) the meeting was not audio recorded, and we were relying on the notes with the risk of losing or missing certain information.; (3) we cannot reject the hypothesis that participants in our study were compassionate people because the compassion was not evaluated before the meetings.

### Recommendations for policy implementation and further research

The current study revealed that when people with dementia get together, they have compassion for each other. They were not only the care recipients but may even become compassionate by living with dementia. As stated in the introduction section, caregivers received more emphasis than people with dementia in the Japanese national dementia strategy. Of course, the burden of caregivers is an important issue, however, we prioritized the people with dementia in the national dementia policies, which was followed by care for caregivers.

Further research should conduct such meetings in diverse settings and with people with various backgrounds and analyse the records to generalize our results. In addition to their discourses, it will be necessary to collect the discourse of people around the participants, to evaluate the situation from multiple perspectives.

## Conclusions

People with dementia and older people with subjective cognitive impairment were recruited at the CBPR center for a peer support meeting. There, the challenges of living with dementia were discussed. After analysing the discourse, five overarching categories were extracted: “Experience of living with dementia,“ “Quest of symptoms,“ “Life story,“ “Hope,“ and “Compassion.“ Participants noted not only being cared for but also exchanging information and exploring symptoms and resilience. In addition, more positive aspects of living with dementia, such as “Hope” and “Compassion,“ were discussed. Therefore, peer support meetings that only included people with dementia may positively impact participants. This evidence will be a basis for promoting peer support meetings in Japan in the future. Further research on the discourse of those around the participants is needed to assess the multifaceted nature of this situation.

## Data Availability

The data that support the findings of this study are available on request to the corresponding author, F.M. The data are not publicly available due to restrictions their containing information that could compromise the privacy of research participants.
